# Knowledge, Attitudes, and Practice About Emergency Contraception Among Saudi Women of Childbearing Age of Eastern Region in Saudi Arabia

**DOI:** 10.7759/cureus.49737

**Published:** 2023-11-30

**Authors:** Amira Okud, Humaira Zareen, Hajer AlSaif, Hadeel Alsayil, Maryam Alrashed, Aeshah Alhejji, Maryam BoSaleh, Mariyyah A Almuhaini, Sayed Ibrahim Ali

**Affiliations:** 1 Department of Obstetrics and Genecology, King Faisal University, Alhasa, SAU; 2 College of Medicine, King Faisal University, Alhasa, SAU; 3 Otolaryngology - Head and Neck Surgery, King Fahad Specialist Hospital, Khobar, SAU; 4 Collage of Medicine, King Faisal University, Alhasa, SAU; 5 Family and Community Medicine, King Faisal University, Hofuf, SAU

**Keywords:** eastern region, saudi arabia, oral progestogen, copper iud, unprotected sexual intercourse, emergency contraceptive drugs

## Abstract

Background and aim

Emergency contraception (EC) refers to methods of contraception that are used within 72 hours up to 120 hours after unprotected intercourse to prevent unintended pregnancy. It can postpone ovulation, stopping fertilization. Ella® (progestin receptor modulator), plan B, birth control tablets, and the copper intrauterine device (IUD) are examples of emergency contraception. The aim of the study is to evaluate knowledge, attitudes, and practices of emergency contraception among Saudi women of childbearing age in the eastern province of Saudi Arabia.

Methods

It is a descriptive cross-sectional study conducted in the eastern region of Saudi Arabia. Data was collected through a pretested online questionnaire after approval from an ethical committee and women's consent to participate in the study. Women between 18 and 49 years old were included in the study. Women aged under 18 and over 49 years were excluded from the study to make the sample population more homogenous. Participants were provided a link to a questionnaire form to be completed from their devices (phone or laptop).

Results

A total of 648 childbearing Saudi women completed the survey. The majority were between 18 and 25 years old. Approximately 263 (40.6%) had no children, and 348 (53.7%) earned less than 5,000 SAR monthly. Four hundred and sixty-seven (72%) of the participants had never used emergency contraception. Four hundred and seventy-eight (73.8%) did not know the maximum acceptable time for using EC. Three hundred and fourteen (48.5%) did not know the potential risks to the baby in case of failure of emergency contraception. Two hundred and twenty-three (34%) patricians wrongly believed that there would be a potential risk to the baby if the patient got pregnant after using emergency contraception. Three hundred and eight (47.5%) participants supported the idea of the availability of emergency contraception without a prescription, and a majority believed that they would not feel shy in asking for emergency contraception. Five hundred and seventy-one (88%) participants did not visit any family planning clinic last year. A significant source of EC information was a doctor or a family planner, 206 (31.8%). A considerable barrier to EC use was fear of side effects and health risks, as reported by 382 (59%) respondents.

Conclusions

The current study reveals that participants have positive attitudes towards emergency contraception, but use is deficient because of poor knowledge and lack of awareness. Our study urges the urgent need for awareness campaigns by health professionals to improve learning and remove wrong fears and beliefs about emergency contraception.

## Introduction

Emergency contraception refers to methods of contraception that are used within 120 hours up to 72 hours after unprotected intercourse to prevent unintended pregnancy [1]. Even though there are a variety of methods of contraception, and it's broadly assumed that contraception knowledge has increased, particularly in the Western world, the rate of unintended pregnancy is estimated to be 85 million worthwhile [2]. Unwanted pregnancy occurs when one or both parents do not want to conceive despite using contraception [3]. Furthermore, it was discovered that emergency contraception is linked to lowering risks of unsafe abortion, HIV transmission from mother to newborn, and increased maternal mortality rates, encompassing conditions like pre-eclampsia, postpartum hemorrhage, and postpartum pre-eclampsia [2]. 

Emergency contraception provides excellent safety, with no death or serious complications [4]. There are different interventions available in the world, including (1) ulipristal acetate (UPA) under the brand of Ella®, an oral progesterone receptor agonist-antagonist, used up to 120 hours after unprotective intercourse; (2) levonorgestrel (LNG) used up to 72 hours; (3) the copper intrauterine device (Cu-IUD) branded as ParaGard® T-380, which is the most effective EC that used for women above 35 years old and is used up to 120 hours; (4) combined oral contraceptives which are documented to be the least effective EC and have not proved to reduce rates of unintended pregnancy or abortion [5-7]. Hormone-based drugs, such an oral progesterone receptor agonist-antagonist, work by limiting the release of eggs or preventing a fertilized egg temporarily from implanting in the uterus, while copper IUD is placed by healthcare workers, occupying the fundus to avoid embryo implantation inside the fundus [8,9]. The US Food and Drug Administration also showed no evidence that EC harms an established pregnancy [10].

Based on previous studies, the rate of knowledge of EC in Saudi Arabia was only 6.2% [11]. A study conducted among Saudi women of childbearing age assessing their knowledge and attitudes concluded that only 16.8% claimed they knew about EC, and even though there is a lack of knowledge, there is a positive attitude toward the future use of EC [12].

Moreover, the study revealed that only nine women out of 370 respondents, representing 2.4%, visited a family planning clinic the previous year and knew about EC. Several factors could be attributed to the lack of adequate knowledge about EC, including family members as the primary source of information, besides social media, and friends as other sources [13]. Additionally, the role of health workers in providing information about contraception was limited, and family planning services were rarely a source of information regarding EC. Additionally, to respect the cultural norms of Saudi Arabia, physicians avoid discussing sexual issues in their clinical practices.

Research studies in sub-Saharan Africa showed that the use of EC is low, ranging from 0% in the Democratic Republic of the Congo and Ethiopia to 54.1% in Nigeria [14]. Based on a study done by Alharbi et al. in 2019 among women of childbearing age in Saudi Arabia, only 37.1% of participating women with knowledge had used EC [11]. Another research conducted in Al-Qassim, Saudi Arabia, stated that only 44.8% were using or had used a contraceptive method continuously for at least one year. Oppositely, there is an increased prevalence of EC usage in Iraq [15]. Awareness about EC, religion, and monthly income are the main factors influencing the usage of EC. Even though there is low usage of EC, there is a positive attitude toward EC [16].

The current study aims to determine the knowledge, attitudes, and practice of EC among Saudi women of childbearing age in the eastern region of Saudi Arabia. Previous studies in Saudi Arabia indicated insufficient knowledge among women regarding EC; counseling represented 16.8% of participants, showing an increase from the 6.2% reported by Karim et al. in 2015 [11,15]. We expect that highly educated women and those who are working know more about EC. Therefore, the current study attempts to increase the knowledge of EC to increase awareness of the risk of unwanted pregnancy. That can be done by encouraging the utilization of EC and providing information about EC benefits and the proper use of them [11,17].

## Materials and methods

Methods

A cross-sectional descriptive study was conducted using a survey questionnaire in the eastern region of Saudi Arabia among women of childbearing age of 18-49 years. Women under 18 and over 49 years were excluded from the study to make the sample population more homogenous.

The sample size was calculated using the formula, n= (z)^2^p(1-p)/d^2^, with a confidence level of 95%, an estimated proportion of 50%, and a 5% level of precision; the appropriate sample size was calculated to be 385. The total received responses was 648.

The questionnaire administered online was created by Alharbi et al. [13] andconfirmed by Amira Okud Mohammed Osman. The questionnaire is composed of two parts: the first part was gathering data on socio-demographic characteristics, while the second consisted of questions to evaluate participants' knowledge, attitudes, and practices about emergency contraception.

Data collection

Data was collected through an online questionnaire via Google Drive(Google, Inc., Mountain View, US). The questionnaire was pilot-tested by 30 participants to identify potential weaknesses. Once the review process ended, the final version was launched. Participants were provided a link to a questionnaire form to be completed from their devices (phone or laptop).

Ethical aspects

Anonymity and confidentiality were guaranteed at all times when answering the questionnaire. Before completing the questionnaire, participants were informed about the study's aim and were asked for their agreement to participate in the survey. The participants provided all of the information. Clearance from the ethical committee was obtained from the dean of scientific research at King Faisal University on 14/12/2021 (KFU-REC-2021-DEC-EA000265) with a validity of 24 months.

Data analysis

Exploratory data analysis was carried out to detect any potential outliers. The analysis considers the study's targeted group. Numbers, percentages, the mean, and standard deviation were used to present the descriptive statistics. The chi-square test examined the association between knowledge, attitudes, and practices concerning EC with regard to the women's socio-demographic factors. A p-value of 0.05 was considered as statistically significant. The Statistical Package for Social Sciences (SPSS) version 26 was used to analyze the data (IBM Inc., Armonk, US).

## Results

The study was carried out to evaluate the women's knowledge, attitudes, and practices about emergency contraception. Six hundred and forty-eight childbearing Saudi women responded to the survey questionnaire.

Table 1 shows the socio-demographic characteristics of the women. Three hundred and fifty-eight (55.2%) respondents were between 18 and 25 years old (mean age was 23 years), with nearly half being students, 299 (46.1%). Women who were university students constituted 332 (51.2%) of the sample. Two hundred sixty-three (40.6%) respondents had no children, and 348 (53.7%) earned less than 5,000 SAR monthly.

**Table 1:** Socio-demographic characteristics of the childbearing Saudi women (n=648)

Study variables	N (%)
Age group	
18 – 25 years	358 (55.2%)
26 – 35 years	148 (22.8%)
36 – 49 years	142 (21.9%)
Occupational status	
Housewife	199 (30.7%)
Student	299 (46.1%)
Employed	129 (19.9%)
Retired	21 (03.2%)
Educational level	
Primary	15 (02.3%)
Secondary	109 (16.8%)
University student	332 (51.2%)
University graduate	164 (25.3%)
Postgraduate	28 (04.3%)
Number of children	
None	263 (40.6%)
One child	112 (17.3%)
Two children	80 (12.3%)
More than two children	193 (29.8%)
Monthly income (SAR)	
<5000	348 (53.7%)
5000 - 10000	160 (24.7%)
>10000	140 (21.6%)

The knowledge, attitudes, and practices towards emergency contraception are shown in Table 2. Four hundred and sixty-seven (72%) participants had never used emergency contraception. Four hundred and seventy-eight (73.8%) participants did not know the maximum acceptable time for using EC pill and IUD after sexual intercourse. The potential risks to the baby in case of failure of emergency contraception were not known by 48.5% of the respondents. Two hundred and twenty-three (34%) patricians wrongly believed that there would be a potential risk to the baby if the patient got pregnant after the use of emergency contraception. Three hundred and eighty-one (58.8%) participants replied that advertisements of emergency contraception must be at a larger scale for better awareness. Three hundred and eight (47.5%) participants supported the idea of the availability of emergency contraception without a prescription, and the majority believed that they would not feel shy in asking for emergency contraception. Five hundred and seventy-one (88%) participants did not visit any family planning clinic last year. Four hundred and forty-four (68.5%) women replied that both male and female partners should decide on using EC (see Table 2).

**Table 2:** Assessment of knowledge, attitudes, and use of emergency contraception (n=648) *Indicates correct answer IUD - intrauterine device; EC - emergency contraception

Statements	N (%)
Knowledge statements	
Have you ever used emergency contraception?	
Yes*	181 (27.9%)
No	467 (72.1%)
What is the maximum acceptable time for using EC (pill) after sexual intercourse?	
Immediately after sex	68 (10.5%)
In the first 48 hours	122 (18.8%)
Within 72 hours*	61 (09.4%)
Within 5 days	36 (05.6%)
I don't know	361 (55.7%)
What is the maximum acceptable time for using EC (IUD) after sexual intercourse?	
Immediately after sex	21 (03.2%)
In the first 48 hours	53 (08.2%)
Within 72 hours	29 (04.5%)
Within 5 days*	67 (10.3%)
I don't know	478 (73.8%)
Is there a potential risk to the current pregnancy if emergency contraception is used?	
There is a risk	225 (34.7%)
There is no risk*	109 (16.8%)
I don't know	314 (48.5%)
Level of knowledge	
Without knowledge	345 (53.2%)
With knowledge	303 (46.8%)
Attitudes statements	
EC reduces pregnancy by up to 75%. Will you continue to use this type of contraceptive method?	
Yes	197 (30.4%)
No	228 (35.2%)
Unsure	223 (34.4%)
Should emergency contraceptives be advertised more widely?	
Yes	381 (58.8%)
No	111 (17.1%)
Unsure	156 (24.1%)
Should emergency contraception be available without a prescription?	
Yes	308 (47.5%)
No	243 (37.5%)
Unsure	97 (15.0%)
Would you feel shy to ask for EC?	
Yes	126 (19.4%)
No	425 (65.6%)
Unsure	97 (15.0%)
Practice statements	
Did you visit a family planning clinic last year?	
Yes	77 (11.9%)
No	571 (88.1%)
Who decides the use of EC	
Female partner	199 (30.7%)
Male partner	05 (0.80%)
Both	444 (68.5%)

**Figure 1:**
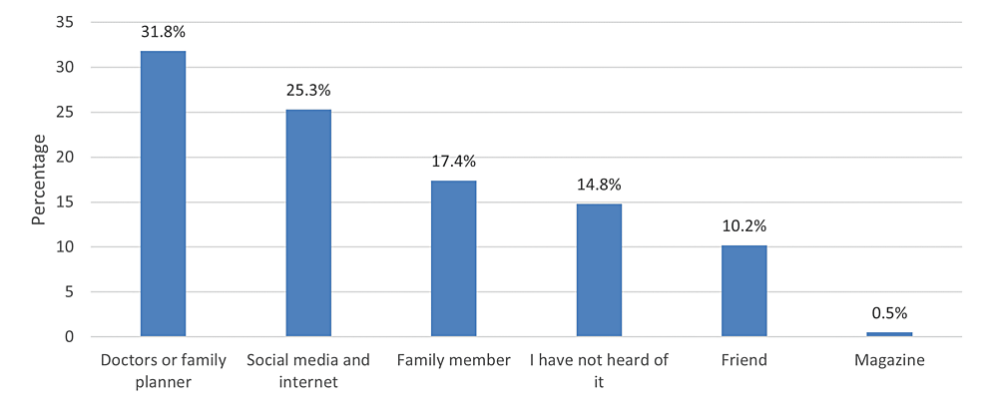
Source of information about emergency contraception

**Figure 2:**
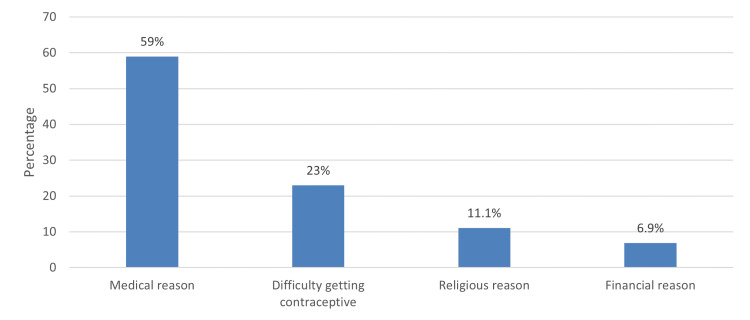
Barriers to using emergency contraception

A significant source of EC information was a doctor or a family planner, 206(31.8%), while the least prevalent information source was a magazine, 3 (0.5%; see Figure 1). A significant barrier to EC use was fear of side effects/health risks reported by 382 (59%) of respondents, followed by difficulty in getting contraceptives, 149(23%), and religious reasons as reported by 72 (11.1%) of participants (see Figure 2).

Table 3 shows the relationship between the knowledge of EC and the socio-demographic characteristics of participants. Knowledge about EC was significantly higher among the oldest age group (p=0.001), those who were employed (p<0.001), having two or more children (p<0.001), having more than 10,000 SAR of monthly income (p=0.002), and having social media and internet as the sources of EC information (p<0.001). No significant differences regarding EC knowledge were noted regarding education (p=0.228) and barriers to using EC (p=0.583).

**Table 3:** Relationship between the knowledge of emergency contraception according to the socio-demographic characteristics of childbearing Saudi women ^§^ p-value was calculated using the chi-square test; ** significant at p<0.05 level EC - emergency contraception

Factor	Level of knowledge		P-value ^§^
	Without knowledge: N (%), n=345	With knowledge: N (%), n=303	
Age group			
18 – 25 years	213 (61.7%)	145 (47.9%)	0.001 **
26 – 35 years	74 (21.4%)	74 (24.4%)	
36 – 49 years	58 (16.8%)	84 (27.7%)	
Occupational status			
Unemployed	106 (30.7%)	114 (37.6%)	<0.001 **
Student	184 (53.3%)	115 (38.0%)	
Employed	55 (15.9%)	74 (24.4%)	
Educational level			
Secondary or below	60 (17.4%)	64 (21.1%)	0.228
University or higher	285 (82.6%)	239 (78.9%)	
Number of children			
None	179 (51.9%)	84 (27.7%)	<0.001 **
One child	48 (13.9%)	64 (21.1%)	
Two or more	118 (34.2%)	155 (51.2%)	
Monthly income (SAR)			
<5000	208 (60.3%)	140 (46.2%)	0.002 **
5000 - 10000	73 (21.2%)	87 (28.7%)	
>10000	64 (18.6%)	76 (25.1%)	
Source of information			
Magazine	01 (0.50%)	02 (0.40%)	<0.001 **
Friend	17 (08.6%)	49 (10.9%)	
Family member	35 (17.8%)	78 (17.3%)	
Doctors or family planners	89 (45.2%)	117 (25.9%)	
Social media and the Internet	35 (17.8%)	129 (28.6%)	
Not heard	20 (10.2%)	76 (16.9%)	
Barriers to using EC			
Religious reason	20 (13.4%)	14 (09.0%)	0.583
Medical reason	88 (59.1%)	92 (59.0%)	
Financial reason	10 (06.7%)	11 (07.1%)	
Difficulty getting contraceptive	31 (20.8%)	39 (25.0%)	

Table 4 shows the relationship between the continuous use of EC and the socio-demographic characteristics of women. Continued use of EC was significantly more common among women with two or more children (p=0.004) and those who indicated doctors or family planners as the sources of EC information (p<0.001). No significant relationship was found between continuous use of EC and age, occupation, education, monthly income, and barriers to using EC (all p>0.05).

**Table 4:** Relationship between the attitude toward emergency contraception according to the socio-demographic characteristics of childbearing Saudi women ^§^ p-value was calculated using the chi-square test; ** significant at p<0.05 level EC - emergency contraception

Factor	Continuous use of the contraceptive method		P-value ^§^
	Yes: N (%), n=197	No/unsure: N (%), n=451	
Age group			
18 – 25 years	98 (49.7%)	260 (57.6%)	0.164
26 – 35 years	49 (24.9%)	99 (22.0%)	
36 – 49 years	50 (25.4%)	92 (20.4%)	
Occupational status			
Unemployed	69 (35.0%)	151 (33.5%)	0.145
Student	81 (41.1%)	218 (48.3%)	
Employed	47 (23.9%)	82 (18.2%)	
Educational level			
Secondary or below	38 (19.3%)	86 (19.1%)	0.948
University or higher	159 (80.7%)	365 (80.9%)	
Number of children			
None	65 (33.0%)	198 (43.9%)	0.004 **
One child	30 (15.2%)	82 (18.2%)	
Two or more	102 (51.8%)	171 (37.9%)	
Monthly income (SAR)			
<5000	102 (51.8%)	246 (54.5%)	0.293
5000 - 10000	45 (22.8%)	115 (25.5%)	
>10000	50 (25.4%)	90 (20.0%)	
Source of information			
Magazine	01 (0.50%)	02 (04.0%)	<0.001 **
Friend	17 (08.6%)	49 (10.9%)	
Family member	35 (17.8%)	78 (17.3%)	
Doctors or family planners	89 (45.2%)	117 (25.9%)	
Social media and the Internet	35 (17.8%)	129 (28.6%)	
Not heard	20 (10.2%)	76 (16.9%)	
Barriers to using EC			
Religious reason	07 (06.5%)	27 (13.6%)	0.08
Medical reason	61 (57.0%)	119 (60.1%)	
Financial reason	11 (10.3%)	10 (05.1%)	
Difficulty getting contraceptive	28 (26.2%)	42 (21.2%)	

Table 5 shows the relationship between visiting a family planning clinic and the socio-demographic characteristics of the women. There were significantly more family planning clinic visits among those who were currently employed (p=0.019), those with two or more children (p=0.003), those who were earning more than 10000 SAR monthly (p=0.013), and those who indicated doctors or family planners as the sources of EC information (p=0.041). On the other hand, age, educational level, and barriers to using EC did not show a significant relationship with previous visits to a family planning clinic (p>005).

**Table 5:** Relationship between the practice of emergency contraception according to the socio-demographic characteristics of childbearing Saudi women ^§^ p-value was calculated using the chi-square test; ** significant at p<0.05 level EC - emergency contraception

Factor	Visit a family planning clinic		P-value ^§^
	Yes: N (%), n=77	No: N (%), n=571	
Age group			
18 – 25 years	36 (46.8%)	322 (56.4%)	0.094
26 – 35 years	25 (32.5%)	123 (21.5%)	
36 – 49 years	16 (20.8%)	126 (22.1%)	
Occupational status			
Unemployed	26 (33.8%)	194 (34.0%)	0.019 **
Student	27 (35.1%)	272 (47.6%)	
Employed	24 (31.2%)	105 (18.4%)	
Educational level			
Secondary or below	12 (15.6%)	112 (19.6%)	0.399
University or higher	65 (84.4%)	459 (80.4%)	
Number of children			
None	18 (23.4%)	245 (42.9%)	0.003 **
One child	14 (18.2%)	98 (17.2%)	
Two or more	45 (58.4%)	228 (39.9%)	
Monthly income (SAR)			
<5000	30 (39.0%)	318 (55.7%)	0.013 **
5000 - 10000	28 (36.4%)	132 (23.1%)	
>10000	19 (24.7%)	121 (21.2%)	
Source of information			
Magazine	01 (01.3%)	02 (0.40%)	0.041 **
Friend	06 (07.8%)	60 (10.5%)	
Family member	13 (16.9%)	100 (17.5%)	
Doctors or family planners	36 (46.8%)	170 (29.8%)	
Social media and the Internet	13 (16.9%)	151 (26.4%)	
Not heard	08 (10.4%)	88 (15.4%)	
Barriers to using EC			
Religious reason	08 (16.3%)	26 (10.2%)	0.334
Medical reason	29 (59.2%)	151 (59.0%)	
Financial reason	01 (02.0%)	20 (07.8%)	
Difficulty getting contraceptive	11 (22.4%)	59 (23.0%)	

## Discussion

The present study evaluated the women's knowledge, attitudes, and practices about emergency contraception among 648 childbearing Saudi women in the eastern region of Saudi Arabia. In our study, most participants were between 18 and 25 years old with a mean age of 23 years, and nearly half of them were university students. However, the mean age was comparatively higher in a similar study conducted in Riyadh, Saudi Arabia, by Karim et al., which showed the mean age of the participants was 37.85 [15]. Similarly, a study done in Riyadh, Saudi Arabia, by Alharbi et al., showed the mean age of the participants of 32 years [11] Moreover, the findings of this study showed that almost half of the participants were university students. This finding is similar to a survey conducted in Al-Qunfudah, Saudi Arabia showing a prevalence of students of 47% [18]. However, a study conducted in Ghana showed a comparatively lower prevalence of students in the sample (38.14%) [19].

The knowledge about EC in our study was significantly low; only 61 (9.4%) and 67 (10.3%) participants had the correct knowledge about the maximum acceptable time for using EC pill and IUD after sexual intercourse, respectively. Most participants did not know the answer to the questions, respectively 361 (55.7%) and 478 (73.8%) respondents. The finding is similar to the results of the studies conducted in Riyadh, Saudi Arabia, which showed that only 6.2% of the participants claimed that pregnancy could be prevented following unprotected sex, and a study in Kuwait showed that only 9.7% of participants claimed they had prior knowledge about ECs [11,20]. Additionally, a study conducted in Saudi Arabia by Karim et al. [15] showed knowledge about EC by 6.2% of the participants, and a study done in Coimbra, Portugal, showed a comparatively higher level of knowledge, 31.2% [21]. A recent study in Al-Qunfudah, Saudi Arabia, exhibited higher EC knowledge levels (85.25% for contraception pills and 57.75% for IUDs) [19]. Furthermore, a study conducted in India reported a higher level of awareness (92.7%) than previous studies [10]. These findings indicate an improvement in the level of awareness recently in Saudi Arabia. One probable explanation is the accuracy of information about the concept of family planning, which represents a significant source of knowledge in our study [22].

However, 314 (48.5%) of respondents were unaware of the danger or lack thereof associated with pregnancy following emergency contraception. Two hundred and twenty-five (34.7%) responders believed there is a risk in the current pregnancy following EC. Conversely, a study by Yeboahet al. in Ghana [19] reported that most participants (93%) knew the side effects of EC. This study further showed that 197 (30.4%) of the participants had a positive attitude toward using EC if it reduces the pregnancy rate up to 75%. Moreover, 381 (58.8%) of the respondents agreed that EC should be advertised, and 308 (47.5%) agreed that EC should be available without a prescription. Four hundred and twenty-five (65.6%) participants in our study expressed willingness to inquire about EC without hesitation, consistent with findings by Alharbi et al. [11]. These findings are identical to a study done in Al-Qunfudah but much higher, representing almost 85% of the participants with positive attitudes towards EC [18]. Moreover, a study done in Ghana [19] showed that 53% had a positive attitude toward emergency contraception. However, women in Kuwait showed a negative attitude toward emergency contraception, representing only 8 (7.8%). More so, neither would they use EC or recommend them to a friend. In addition, they believed that EC should not be available in health centers [20].

Considering the use of EC, 467(72%) of the participants had never used emergency contraception compared to the findings of Malak S Alharbi's study, which showed only (37.1) %. In the present study, most participants did not visit the family planner 571(88.1%), while only 77(11.9%) saw the clinic. This finding is lower than a study conducted in Iraq, which reported a higher prevalence rate (67.3%) of using family planning methods [20]. Consistent with the current study, the finding of a study in Riyadh, Saudi Arabia, showed (2.4%) of their participant with knowledge had visited a family planner [11] this finding is low compared to our result, which hence increases awareness in Saudi Arabia in the fast few years.

This study further showed that the participants' source of information about EC among women with knowledge revealed that doctors and family planners were the primary source of information, 207 (31.8%), followed by social media and the internet, 164 (25.3%). In comparison, the least mentioned source was magazines, 3 (0.5%). That demonstrates the positive effects of healthcare workers on Saudi women, and this is confirmed by another study in Riyadh, Saudi Arabia, in which most participants considered family members a significant source of information (60%) [17]. Similarly, in a study in Al-Qunfudah, Saudi Arabia, their primary source of information was family and friends (38.75%) [18]. Controversially, a study done in Portugal showed the media as the most common source of information (63.4%) [19]. This finding is similar to a study done in India, which revealed that 65.5% of the participants used audio-visual media as a significant source of information [22].

We found in our study that the most common barrier to EC use was harmful health effects noted by 382 (59%) among both women with and without knowledge, followed by difficulty getting contraceptives, 149 (23%), and religious reasons, 72 (11.1%). The study in Riyadh found barriers to EC use were related to medical causes in 48.4% and religious beliefs in 14.5% [11]. Karim et al. reported that barriers to EC use were related to medical consequences in 73.3% and religious beliefs in 13.3% [15]. 

Our findings on the relationship between the knowledge of EC and the socio-demographic characteristics of childbearing Saudi women showed that women with knowing EC were significantly more prevalent among the oldest age group, 84 (27.7%); in comparison, the study conducted in Riyadh showed that the youngest women had a comprehensive understanding and awareness regarding emergency contraception [15]. Furthermore, our findings that those who were employed, 74 (24.4%), had two or more children, 155 (51.2%), and had more than 10,000 SAR of monthly income, 76 (25.1%), are similar to the same previous study, which showed that the knowledge of EC among participants was significantly better among working women, who have had multiple births, and who have had a higher income [13].* *However, there are no significant differences in the level of EC knowledge in terms of education, reported lower utilization of EC among educated women (78.9%). Similarly, the study in Ghana reported lower utilization of EC among educated women (73.77%) [19].

## Conclusions

The current study reveals that participants have positive attitudes towards emergency contraception, but use is limited because of poor knowledge and lack of awareness. Our study urges the need for awareness campaigns by health professionals to improve learning and remove wrong fears and beliefs about emergency contraception.
